# Questioning reliability assessments of health information on social media

**DOI:** 10.5195/jmla.2017.108

**Published:** 2017-01

**Authors:** Nicole K. Dalmer

## Abstract

This narrative review examines assessments of the reliability of online health information retrieved through social media to ascertain whether health information accessed or disseminated through social media should be evaluated differently than other online health information. Several medical, library and information science, and interdisciplinary databases were searched using terms relating to social media, reliability, and health information. While social media’s increasing role in health information consumption is recognized, studies are dominated by investigations of traditional (i.e., non-social media) sites. To more richly assess constructions of reliability when using social media for health information, future research must focus on health consumers’ unique contexts, virtual relationships, and degrees of trust within their social networks.

## BACKGROUND

Health information is increasingly sought through online and socially networked platforms [1], given their constant availability, anonymity, and purported wealth of information [2]. The first generation of e-patients (i.e., those who seek online guidance for their ailments) reported that they found improved health information [3] and indicated that the medical information and guidance that they found online was more complete and useful than what they received from their clinicians [4].

The reliability of health-related information found on the Internet and how this information is used by patients, their caregivers, and other lay health consumers has been a cited issue of concern for health care professionals and policy makers, particularly given the impact that incorrect, vague, or overly technical information can have on the health-related outcomes of those searching the web [5–7]. A sizeable number of studies have previously examined and reported on both the benefits and the risks of health-related information retrieved from online sources [8–14]. One study found that many people searching online for health information trusted the advice and resources that they found [15], whereas 70% of studies in a systematic analysis of health website evaluations found that quality was a problem on the Internet [16]. Over an 8-year period, a survey found a substantial increase both in participants’ use of and confidence in the health information they found online [17]. An increased use of online health information and an increased sense of trust in that information highlights the complex challenges and opportunities of assessing online health information, particularly when consumers are using this information to make decisions about their own and their families’ and friends’ health and well-being.

“Social media has become an indelible part of the public health landscape” [18], with participation and membership in social media sites more than quadrupling between 2005 and 2009 across age, education, and ethnicity [19]. Social media outlets are increasingly being used to access, share, and disseminate health information and are recognized as having the potential to reshape health care. Social media tools allow for more democratic and less hierarchical communication networks for both those accessing and disseminating health-related information [20]. Previous research has examined consumer trust in online health information [7], patients’ evaluation and use of online health information [21], and online health information reliability [6, 22, 23]. However, despite the accelerated uptake and use of social media for health information, there has yet to be an exploration of lay health consumers’ assessments of the reliability of health information accessed or shared through social media sites. While Moorhead et al. [24] and Antheunis et al. [25] found that reliability is a concern for those who use social media to access and communicate health information, there is a lack of scholarship that either attempts to address this concern or that proposes amendments to established definitions of reliability. In alignment with Witteman and O’Grady’s [26] recommended future e-Health 2.0 research directions in examining how consumers assess the reliability of Web 2.0 sites, the present review aims to address this gap in the literature, examining lay health consumers’ conceptualizations of online health information credibility to determine whether the increased use of social media to access, manage, and share health information requires a new lens or different criteria to evaluate the reliability of this information.

To begin to answer whether health information disseminated through social media needs to be evaluated differently than other online health information, this narrative review, though nonsystematic, sought to synthesize a variety of studies into a comprehensive overview [27], providing both guidance and a prompt for further scholarship in this area. A narrative review was chosen given that one of its main strengths is its ability to provide an overview of heterogeneous research produced on a certain topic [27].

## ESTABLISHING KEY CONCEPTS

To allow a thorough examination of lay health consumers’ perceptions of online health information retrieved via social media sites, the central concepts must first be established and outlined. Reliability as it relates to information is a complex concept. According to Adams, reliability concerns three aspects: (1) the technical components of websites and Internet-based applications, (2) information content, and (3) expected end-user behavior [6]. Adams also notes that in the literature, reliability is often used interchangeably with terms such as quality, credibility, trustworthiness, and accuracy [6]. Credibility is also noted as an interplay between receiver, source, and message characteristics [23]. Social media, also sometimes called Web 2.0 or the socially networked web, is defined as a tool that must meet at least two of three conditions: (1) it must allow people to communicate, collaborate, and build community online; (2) it can be shared, reused, or remixed; and (3) it allows people to learn easily from and capitalize on the behavior or knowledge of others [28]. Medicine 2.0 or Health 2.0 are terms related to social media that imply openness and transparency [29], emphasizing the leveling effect of the Internet, such that regardless of professional experience or knowledge, anyone can share information about health and medical information. Lastly, lay health consumers are individuals who are not health professionals and who use or seek out health information.

## UNDERSTANDING RELIABILITY MARKERS OF HEALTH WEBSITES

Reliability, quality, and accuracy of online health information is important to a vast majority of Internet users, with 86% of online health information users concerned about the veracity of information on the Internet [2]. Studies have reported that the following factors increase users’ trust in and perceptions of reliability in health websites: information completeness [30]; clear navigation [31]; author credentials [32]; similarity of information [33]; professional layout, understandability, professional writing, and citation of scientific references [34]; and hosting by reputable or expert organizations [21]. Although reliability assessments can be highly situational, demographics and the degree of Internet use are reported to influence perceptions of the credibility of web-based information [35].

Recognizing the need to give order to and regulate the quality of online health information, the Health On the Net Foundation created “The Code of Conduct for Medical Websites” (HONcode) in 1996 [36]. While the eight principles of the HONcode (including confidentiality, referencing, and evidence-based information) have merit, there has been little follow-up with this code, which perhaps indicates the difficulty in asserting control over the rapidly expanding and increasingly socially networked web. Certain organizations, such as the American Medical Association, have begun to create their own guidelines for ensuring the accuracy and reliability of their own websites [31], exemplifying the trend of assessing the credibility of online health information primarily at a website level. Given the situational nature of credibility, however, a few studies have considered the importance of the contextual and social surroundings of individuals searching through health websites. Ye for example, reported that consumers’ trust in online health information was not correlated with personal capital such as income, education, and health status [7]. Similarly, Case found that access concerns override issues of quality and credibility of sources for most individuals [37]. Dervin called to attention the value that individuals placed on the helpfulness of the information, “no matter how alarming their inattention to information authority, they mostly care not where the information comes from but whether it is helpful” [38] (p. S79).

Researchers have posited many other factors that are likely to govern the extent to which people feel that they can trust online health information: attractive and professional design, presence or absence of features such as a trust seal, and perceived competence or integrity of the site [21]. In recognition of more individualistic behaviors in assessing information credibility, a small number of studies emphasize the importance of reputation, transparency, and familiarity [39], as well as construction with personal appeal [40], as significant elements in forming reliability judgments about online health information, aptly capturing the emergence of the social life of health information [41].

## ASSESSING RELIABILITY ON SOCIAL MEDIA SITES: PRESENT CONTEXTS

Studies detailing lay health consumers’ assessments of online health information credibility continue to be rooted in traditional health websites, not social media. Indeed, while examinations of credibility assessments of health information retrieved through traditional websites and online resources are abundant in the literature, only a few studies have investigated how lay health consumers assess the reliability of health-related information that they find on social media sites. Despite Adams’s observation that reliability concerns and issues expressed about the general web over the last fifteen years have rematerialized with the emergence of Web 2.0 tools and sites [42], few studies have examined the impact of the increasing adoption of social media as a source of health information on lay health consumers’ assessments of reliability.

The complexity of creating credibility criteria that holistically account for lay health consumers’ varying degrees of trust and integration in social networks may explain the lack of literature on this topic. While there is an established understanding of social media’s increasing role in health information consumption and distribution [43–45], a majority of studies are either descriptive in nature—documenting the considerations, benefits, and potential shortcomings of individuals’ reliability assessments of health-related social media use [29, 46–49]—or focus on social media as a communication tool between patients and health care professionals [50–52].

The rise in social media as a tool for accessing and disseminating health-related information may be in part explained by online users’ changing interaction with information, evolving from simple searching to a now dynamic and collaborative engagement with information [53]. Indeed, social media sites encourage health consumers to communicate and share knowledge directly with one another, fostering interaction, participation, engagement, and community. This is especially visible in the use of social media for support for and education about health-related ailments and concerns [54–56]. As social media sites promote the creation of user-generated content, they blur boundaries between producers and consumers of online health-related information. The rapid growth of peer-to-peer social media adoption also brings about a shift in social interactions, moving from interactions between smaller groups of individuals to community-wide networks in which information can be created, searched, and shared [18].

Part of the uniqueness and utility of social media sites lies in its users’ ability to contribute information online without any specialized knowledge of web language or formatting. Furthermore, social media outlets allow users to choose their degree of participation and involvement or, conversely, their degree of passivity when using Facebook, Twitter, YouTube, blogs, and so on. Lober and Flowers outline a key reason for social media’s rapid and extensive penetration into individuals’ health behaviors: its “ability to turn communication into interactive dialogue” [53] (p. 176). This is underscored by Eytan et al., who indicate social media’s success lies in its ability “to facilitate talking as well as listening, consuming as well as participating” [57] (p. 73).

This unique ability of social media to foster a sense of community and interactivity serves to bring users closer together [19], perhaps owing to social media’s ability to transform health information from static sources of information to interactive communication channels. Lay health consumers use online health communities to seek out second opinions on treatment options, diagnosis information, and experience with health care providers [53]. The same authors found that individuals use social media to share their experiences, reach out for information and opinions, and engage with peers and providers [53]. As social media’s features emphasize flexibility of access, interaction, mobility, multimedia, participation, informality, and feedback [58], the conversational language and more relaxed, interactive environment often found in social media sites may explain this platform’s increasing use as a means to seek out and share health information.

Adams proposes specific areas of concern that researchers may need to consider in examining assessments of the reliability of online health information accessed through social media tools or sites, including disclosure of authorship, information quality, anonymity, and privacy and whether individuals can discern the nature, source, and intention of health information from Web 2.0 tools [42]. While these concerns encompassing the interpretation and assessment of reliable Web 2.0 health information remain, they fail to fully underscore findings that studies of information reliability neglect to consider that information must be applied to individuals’ unique contexts [5]. With the responsibility of information use (and sharing, management, integration, and so on) increasingly placed on individuals, they are expected to not only understand rapidly changing health information, but also to weigh and evaluate potentially conflicting medical information. This need to filter is likely only exacerbated by social media’s ability to display video and other multimedia alongside text. In addition to the content of information sought, information professionals must consider the contexts and situations surrounding individuals who are seeking health information, placing their information needs in the broader context of their unique experiences and surroundings.

## PROMPTING CHANGE: FUTURE DIRECTIONS FOR SOCIAL MEDIA RELIABILITY ASSESSMENTS

Traditional means of assessing the credibility of online health information from conventional websites do not appear to adequately account for the social or relational aspects that play a significant role in social networks or Web 2.0 tools. Socially mediated reliability assessments of online health information present a very complex case in attempting to find criteria that adequately capture both individuals’ situations as well as the context of the relations of the community or network in which each individual is embedded. Not only are health care professionals or information professionals removed as gatekeepers to health information or as “experts” in assessing information’s veracity before that information is shared with lay health consumers, but the peer-to-peer nature of social media also leaves information to be shared and passed to other networked individuals with an unpredictable speed and pattern, making it difficult to track down or correct unreliable health information.

As social software primarily exists on content created by its users, it also blurs the boundaries between producers and consumers of online health-related information, tampering with traditional judgments of professional and amateur online authority and expertise [26]. This complexity in formulating holistic and representative criteria that encompass both the parts and the whole of social interactions may explain the dearth of retrievable literature on the topic of lay health consumers’ reliability assessments of health information retrieved through social media sites.

An understanding of social media and social networks reveals, however, that assessments of online health reliability retrieved or shared through Web 2.0 tools are more complex than simply taking into consideration an individual’s situation or context. Social media sites are conducive for collaborative and crowdsourced thinking, allowing for potentially prolific information production and sharing [59]. In these social media sites, individuals are participants in virtual communities and networks. Consequently, reliability assessments of health information located on social media sites must not only take into account individuals’ contexts and situations, but must also take into account the community dynamics in which they are situated and connected.

As Kadushin states, “Social participation produces social networks. Participation also leads to trust and to a general culture of trust which in turn promotes participation in a virtuous circle” [60]. This cycle was exemplified in Marton’s research [61], which found that users who perceived the health information on web-based communication sites to be reliable were not only inclined to regard the content on these sites as highly relevant, but also spent more time using these sites. In determining how to build tools or criteria to best assess whether health-related information retrieved through a social media site can be trusted or is reliable, a decreased focus on more traditional assessments of reliability (such as specific facets of the information or site itself, including logos or crests on a website, professional layout, and so on) and an increased focus on social aspects, such as relationships and trust between the actors on social media sites or networks, may be prudent.

As social media use and social network participation continue to increase, will assessments and perceptions of online health-related information reliability shift from a focus on information as the primary object examined to a focus on either the level of trust between the individuals sharing the information or on the individual who is producing, sharing, or recommending health-related information? Conceivably, reliability assessments of health information on social media sites may become a matter of the trustworthiness of the individuals disclosing or seeking information, not a matter of the content, features, or presentation of the information itself. Future studies may elect to examine whether the degree of closeness or trustworthiness between individuals on a social media site or the degree of closeness or trustworthiness of an online or virtual community modulates or is correlated with the degree to which the reliability of the information itself is appraised. For example, are members of a close-knit virtual community or Facebook group less likely to question the veracity of health-related information shared through their networks, owing to the sense of trust already present among its members?

In light of the established importance of the social and relational aspects of reliability assessments for health information retrieved through social media sites, an amendment to the established definitions of credibility outlined at the onset of this narrative review may be necessary. Adams aptly notes that “social networking and the shift from text-based information to symbolic information, images or interactive information, are considered to enhance patient education and to provide opportunities to reach diverse groups of patients” [42]. As such, social media’s capacity to change the way in which online health information is produced, accessed, trusted, and filtered warrants a greater and more focused examination by information professionals. The evolution from “searching for information, to sharing information, and now to engaging with information” [53] is evidence that an alternate approach, focused on relationships, to understanding lay health consumers’ assessments of the reliability of health information shared or located on social media venues is needed. A shift from examining specific or technical features of a website to an assessment that captures the importance and influence of relational and crowdsourced elements of the social web is necessary for a more accurate understanding of reliability assessments.

When specifically examining reliability assessments of health information retrieved through social media sites, Adams’s three-pronged reliability definition [6] may need to emphasize the end-user behaviors and include a greater community dynamic element, recognizing that the end user is embedded and operates within a network. The definition discussed by Wathen and Burkell [23] may need to expand upon receiver and source characteristics to accentuate the vital role that trust among members of social media sites or social networks plays in modulating assessments of credibility of information shared, recommended, or passed along. Eysenbach’s vision of modeling “social relationships and information concerning ‘who said what about a specific website’ as one promising way to guide consumers to high quality information” [29] may be a promising starting point in attempting to better assess the reliability of health information on social media, while acknowledging the influence of the networked web on collaborative filtering and information sharing.
